# A novel small molecule inhibitor of *Candida albicans* biofilm formation, filamentation and virulence with low potential for the development of resistance

**DOI:** 10.1038/npjbiofilms.2015.12

**Published:** 2015-08-12

**Authors:** Christopher G Pierce, Ashok K Chaturvedi, Anna L Lazzell, Alexander T Powell, Stephen P Saville, Stanton F McHardy, Jose L Lopez-Ribot

**Affiliations:** 1 Department of Biology, South Texas Center for Emerging Infectious Diseases, The University of Texas at San Antonio, San Antonio, TX, USA; 2 Department of Chemistry, Center for Innovation in Drug Discovery, The University of Texas at San Antonio, San Antonio, TX, USA

## Abstract

**Background/Objectives:**

*Candida albicans* is the principal causative agent of candidiasis, the most common fungal infection in humans. Candidiasis represents the third-to-fourth most frequent nosocomial infection worldwide, as this normal commensal of humans causes opportunistic infections in an expanding population of immune- and medically compromised patients. These infections are frequently associated with biofilm formation, which complicates treatment and contributes to unacceptably high mortality rates.

**Methods:**

To address the pressing need for new antifungals, we have performed a high-content screen of 20,000 small molecules in a chemical library (NOVACore) to identify compounds that inhibit *C. albicans* biofilm formation, and conducted a series of follow-up studies to examine the *in vitro* and *in vivo* activity of the identified compounds.

**Results:**

The screen identified a novel series of diazaspiro-decane structural analogs that were largely represented among the bioactive compounds. Characterization of the leading compound from this series indicated that it inhibits processes associated with *C. albicans* virulence, most notably biofilm formation and filamentation, without having an effect on overall growth or eliciting resistance. This compound demonstrated *in vivo* activity in clinically relevant murine models of both invasive and oral candidiasis and as such represents a promising lead for antifungal drug development. Furthermore, these results provide proof of concept for the implementation of antivirulence approaches against *C. albicans* and other fungal infections that would be less likely to foster the emergence of resistance.

## Introduction

*Candida albicans* is generally acquired early in neonatal life and becomes a common commensal of the human oral, vaginal and gastrointestinal tracts, where it normally causes little or no damage to the host. However, as an opportunistic pathogenic fungus of humans, *C. albicans* is also capable of causing a variety of infections ranging from mucosal to life-threatening systemic candidiasis.^1,2^ These infections are an increasing health threat to immune- and medically compromised individuals.^2^ Of all fungal invasive infections, by far candidiasis remains the most common, now representing the third-to-fourth most frequent nosocomial infection in hospitals in the United States and worldwide.^3–6^

A trait that complicates the treatment of *C. albicans* infections in an increasing number of patients is its ability to form biofilms, microbial communities attached to biological or inert surfaces.^7–10^ Biofilm formation occurs on both biological and inert surfaces, as different types of biomaterials support biofilm formation by *Candida*, most notably intravascular catheters.^9,10^ Biofilm formation carries important negative consequences as it leads to high levels of resistance to most clinically used antifungal agents, provides a safe haven for fungal cells and serves as a reservoir for recurring sources of infections.^9,10^ As such, biofilm formation represents one of the major virulence factors contributing to the pathogenesis of candidiasis.

Invasive candidiasis carries unacceptably high mortality rates, ~40–60%, even with treatment using available antifungals,^11^ which underscores the urgent need for the development of new antifungal agents, particularly those with novel mechanisms of action. However, fungi such as *C. albicans* are eukaryotic and there is a paucity of selective pathogen-specific targets that can be exploited for antifungal drug development.^12–14^ Thus, in stark contrast with antibacterial antibiotics, the current arsenal of antifungal drugs is exceedingly short. Moreover, the efficacy of antifungal agents is limited because of toxicity issues and, more recently, the emergence of resistance.^15,16^ Because of its important role during the pathogenesis of candidiasis and its infection, we posited that *C. albicans* biofilm formation represents a high-value target, yet clinically unexploited, for the development of novel antifungal therapies. Here we report on the results of a large-scale chemical library screen to identify small molecule compounds present in a commercially available library, NOVACore, resulting in the identification of a novel hit series of diazaspiro-decane structural analogs with inhibitory activity against *C. albicans* biofilm formation. Further characterization of the activity of the leading candidate from this series, both *in vitro* and *in vivo*, indicates that it has the potential to be developed as an antivirulence compound for the treatment of *C. albicans* infections

## Materials and methods

### Chemical library

We screened a total of 20,000 small molecule compounds present in the NOVACore library from Chembridge Corporation (San Diego, CA, USA), a research-intensive, medicinally relevant chemical library intended to accelerate the hit-to-lead process with built-in structure–activity relationship capabilities. The key properties of compounds in this library are novelty, diversity and ease of follow-up, and small molecules in this library generally have favorable pharmacological characteristics and meet very stringent ‘drug-like’ properties. Compounds in this library were provided as individual stock solutions in 96-well microtiter plates in 10 mM concentrations in dimethyl sulfoxide (DMSO), and the plates are bar-coded for automation and rapid hit identification. Before screening, the compounds were diluted to 0.1 mM by pipetting 2 μl of this concentrated solution into 198 μl of sterile phosphate-buffered saline (PBS; 10 mM phosphate buffer, 2.7 mM potassium chloride, 137 mM sodium chloride (pH 7.4; Sigma, St Louis, MO, USA)) using the wells of presterilized, polystyrene, round-bottom, 96-well microtiter plates (Corning Incorporated, Corning, NY, USA) and stored as working stock solutions at −20 °C. For follow-up experiments, milligram quantities were obtained from stock compounds available for hit resupply from Chembridge Corporation.

### Strains, media and culture conditions

A well-characterized *C. albicans* strain SC5314 (a clinical isolate originally obtained from a patient with disseminated candidiasis^17^) was used primarily in this study. *C. albicans* strain 6482, a clinical isolate obtained from an HIV-infected patient with recalcitrant oropharyngeal candidiasis with high predisposition to develop resistance to multiple antifungal drugs,^18,19^ was also used in serial passage experiments to examine the development of resistance (see below). For routine culture, cells from stocks stored at −80 °C were propagated by streaking onto yeast peptone dextrose (YPD) agar plates (1% (wt/vol) yeast extract, 2% (wt/vol) peptone, 2% (wt/vol) dextrose and 1.5% agar), and incubation overnight at 30 °C. From these, a loopful of cells was inoculated into flasks (150 ml) containing 25 ml of YPD liquid media in an orbital shaker at 180 r.p.m. and grown for 14–16 h at 30 °C. Under these conditions, *C. albicans* grows as budding yeast.

### Screening assay for inhibitors of *C. albicans* biofilm formation

Biofilms of the *C. albicans* wild-type strain SC5314 were formed using the 96-well microtiter plate model previously described by our group.^20,21^ Briefly, cells were harvested from overnight YPD cultures and after washings they were resuspended at a final concentration of 1.0×10^6^ cells/ml in RPMI-1640 supplemented with L-glutamine (Corning) and buffered with 165 mM morpholinepropanesulfonic acid (Sigma). *C. albicans* biofilms were formed on commercially available presterilized, polystyrene, flat-bottom, 96-well microtiter plates (Corning Incorporated). Columns 2 through 11 of the microtiter plate contained 5 μl of compounds previously diluted to render a final concentration of 5 μM. To the first column of the plate, DMSO (Sigma) was added at a concentration equivalent to the concentration in the wells containing compounds (this DMSO concentration is not toxic to *Candida* and does not affect biofilm formation). The final column of the plate remained empty to serve as a background control when the optical density (OD) was determined in a microtiter plate reader (Benchmark Microplate Reader; Bio-Rad, Hercules, CA, USA). The initial screen was performed in duplicate with the plates incubated at 37 °C for 24 h. Following biofilm formation, the wells were washed twice to remove non-adherent cells, and the extent of biofilm formation assessed using semiquantitative colorimetric assay based on the reduction of 2,3-bis(2-methoxy-4-nitro-5-sulfo-phenyl)-2*H*-tetra-zolium-5-carboxanilide (XTT, Sigma) as previously described by our group. The OD of control biofilms formed in column 1 in the absence of compound was arbitrarily set at 100%, and data were calculated as percent biofilm inhibition relative to the average of the control wells.

### Dose–response assays for hit confirmation and determination of potency and toxicity

Compounds identified as hits in the initial screen for inhibitors of *C. albicans* wild-type biofilms were confirmed via dose–response assays. These assays used the same 96-well flat-bottom plate model for inhibition of biofilm formation used in the initial screen, except that the compounds were assayed in serial twofold dilutions at concentrations ranging from 40 to 0.078 μM, with appropriate positive and negative controls. Each compound was assayed in duplicate plates. After incubation (37 °C for 24 h), the microtiter plates were washed and processed using the XTT reduction assay as above. The inhibitory concentration (IC_50_) value, defined as the concentration of each compound leading to 50% inhibition of biofilm formation, was calculated from the results of these assays, using Prism (GraphPad Software, San Diego, CA, USA). In addition, for the examination of activity against preformed biofilms, *C. albicans* SC5314 biofilms were formed in microtiter plates during 24 h as described before (in the absence of compound); the plates were then washed twice with sterile PBS and 100 μl of RPMI containing individual compounds in serial twofold dilutions were added to each test well. The microtiter plates were then incubated at 37 °C for an additional 24 h, washed twice with sterile PBS and processed using the XTT reduction assay.

We used a cell-based assay as an alternative to animal testing to determine the initial toxicity of the selected compounds.^22,23^ Human hepatocellular carcinoma (HepG2) cells (American Type Culture Collection (ATCC)#HB-8065) were maintained in Minimum Essential Medium (Gibco-Life Technologies, Grand Island, NY, USA) supplemented with 10% fetal bovine serum, 1 mM sodium pyruvate (Gibco), 1× Minimum Essential Medium amino-acid solution (Sigma), 100 IU/ml Penicillin and 100 mg/ml Streptomycin (Cellgro). The monolayers of cells were detached using 1×Trypsin/EDTA (Gibco) and centrifuged at 500*g* for 7 m at 4 °C. Cell count was adjusted to 5×10^5^ cells/ml in supplemented Minimum Essential Medium (2×), and 100 μl of the cell suspension was added to each well of white-bottom, 96-well microtiter plates containing 100 μl of serial twofold dilutions of the small molecule compounds (160-1.56 μM) or the DMSO control. The plates were incubated for 24 h at 37 °C, and the number of viable cells was determined using the CellTiter-Glo Luminescent Cell Viability Assay (Promega, Madison, WI, USA). From these data, the CC_50_ value, defined as the concentration of each compound leading to 50% inhibition of cell viability, was calculated using Prism (GraphPad Software).

### Confocal laser scanning microscopy

*C. albicans* strain SC5314 biofilms were grown in six-well plates in the presence or absence of the leading 61894700 compound. Briefly, after 24 h of incubation at 37 °C the resulting biofilms were washed and stained with 25 μg/ml Concanavalin A-Alexa Fluor 594 conjugate (C-11253; Molecular Probes, Eugene, OR, USA) for 1 h in the dark at 37 °C. Confocal laser scanning microscopy was performed with a Zeiss LSM 510 upright confocal microscope (Carl Zeiss, Thornwood, NY, USA), using a Zeiss Achroplan ×40, 0.8-W objective, and a HeNe1 laser with an excitation wavelength of 543 nm. Images of sections in the *xy* plane were taken along the *z* axis, acquired using the resident software and processed using AutoQuant (Media Cybernetics, Bethesda, MD, USA) and IMARIS 6.4 (Bitplane, St Paul, MN, USA).

### Effect on biofilm formation on silicon elastomers

For evaluation of the inhibitory activity against *C. albicans* biofilm formation on silicone, silicone elastomer sheets (Bentec Medical, Woodland, CA, USA) were cut into 1 cm×1 cm squares, cleaned by washing with hand soap and water and autoclaved.^24^ Before biofilm formation, the silicone coupons were incubated in fetal bovine serum overnight at 37 °C. The pieces were washed twice in sterile PBS and placed into the wells of 24-well flat-bottomed presterilized microtiter plates (Corning). To ensure uniform biofilm formation on the entire coupon, 2 ml of a 5×10^6^ cells/ml suspension of *C. albicans* SC5314 in RPMI buffered with morpholinepropanesulfonic acid containing either the leading compound at final concentrations of 2.5, 5, 10 and 20 μM, or no compound (control) was added to the wells containing the silicone pieces and incubated in an orbital shaker at 100 r.p.m. for 2 h at 37 °C. Following this adhesion step, non-adherent cells were removed by washing the silicone pieces twice in sterile PBS and transferring them to wells of a new 24-well plate. Fresh RPMI media containing the compound at the same concentrations were added to the corresponding wells and the plates were incubated for an additional 24 h in an orbital shaker (100 r.p.m.) at 37 °C. After this incubation, the silicone pieces were washed with PBS and processed using the XTT reduction assay, to calculate percent inhibition.

### Effect on *C. albicans* filamentation

*C. albicans* strain SC5314 was grown overnight at 30 °C in YPD liquid media, and 1 in 30 dilutions were made to fresh YPD liquid medium containing 10% serum; these were added to wells in round-bottom microtiter plates containing twofold serial dilutions of the leading 61894700 compound with concentrations ranging from 40 to 0.039 μM. The plates were incubated at 37 °C for 6 h and examined microscopically for differences in cell morphology compared with control wells containing no compound. Microscopy was performed using bright-field light techniques on an inverted microscope equipped for photography (Westover Scientific, Mill Creek, WA, USA).

### Antifungal susceptibility testing under planktonic growth conditions

Planktonic susceptibility testing of *C. albicans* strains against fluconazole, amphotericin B, caspofungin and compound 61894700 was carried out following the Clinical and Laboratory Standards Institute (CLSI) M-27A broth microdilution method, with end points determined at 24 h post inoculation. Besides *C. albicans* SC5314 strain, two additional *C. albicans* clinical isolates (*C. albicans* 11-3478 and 11-3479) provided by the Fungus Testing Laboratory were used as quality controls for antifungal susceptibility testing under these planktonic conditions.

### Serial passage experiments for induction of resistance

Independent initial populations of *C. albicans* were founded from a single colony of *C. albicans* SC5314 and 6482 strains. For each strain, one of these starter cultures was in the YPD medium at 37 °C in the absence of serum (non-filamenting inducing conditions), whereas the other was in RPMI 1640 medium at 37 °C (filament-inducing conditions). For each condition, cultures contained 5 μM of the 61894700 compound, a concentration previously determined to inhibit biofilm formation and filamentation but not to have an effect on overall planktonic growth. Cultures were placed in orbital shakers and every day 10 μl from each culture was serially transferred into 1 ml of the corresponding fresh medium, YPD or RPMI 1640 containing 5 μM of the leading compound, and cells were grown with constant agitation. These daily transfers were performed for 40 days, at which time the concentration of compound was increased to 20 μM, and experiments were continued uninterrupted for an additional 20 days. After daily transfers, population samples were archived in 1 ml of 40% (vol/vol) glycerol at −80 °C. To assess the potential for the development of resistance, cultures grown under filamenting-inducing conditions in RPMI medium in the presence of the small molecule compound were monitored for the presence/absence of hyphae before daily transfers. In addition, cells from populations recovered at different time points were used to seed the wells of microtiter plates, and the ability of the small molecule compound to still inhibit biofilm formation was determined using the same 96-well microtiter plate model methodology as described above for the dose–response assays.

### Determination of the *in vivo* activity of the leading compound in the murine models of hematogenously disseminated candidiasis and oral candidiasis

All animal experiments were performed following the National Institutes of Health (NIH) guidelines and in accordance with the Institutional regulations of Institutional Animal Care and Use Committee (IACUC) in an AAALAC-certified facility at UTSA. Animals were allowed a 1-week acclimatization period before experiments were started. Mice were randomly distributed in different cages and assigned to the different treatment arms, and persons monitoring the animals were not blinded as to the identity of different groups. As per the IACUC guidelines power analyses, using data from similar past experiments, were performed to estimate lower number of animals required for significance.

To prepare the initial infecting inocula cultures of strain SC5314 were grown overnight in YPD broth at 25 °C. Cells were harvested by centrifugation and washed three times with sterile saline. Cells were counted using a hemocytometer and appropriate dilutions of the cells were made in sterile saline for injection. Confirmation of the number and viability of cells present in the infecting inocula was performed by plate count.

For the hematogenously disseminated candidiasis model, a final volume of 200 μl of cell suspensions was injected via the lateral tail vein into 6–8-week-old female BALB/c mice. Groups of mice (*n*=8) were treated intraperitoneally with 0.5 ml of 0.8 mg/ml (corresponding to ~20 mg/kg) of compound 61894700 diluted in 4% DMSO (prepared in saline for injection) 2 days before infection with 3.5×10^5^ cells of *C. albicans* strain SC5314. Treatment continued once a day until 7 days post infection at which time the treatment was stopped. A control group was on the same schedule but received vehicle only injections. To determine the survival curves, days on which the mice died were recorded; moribund mice were humanely killed and recorded as dying the following day. Survival data and differences between groups (treated versus untreated) were analyzed using the Kaplan–Meier and log-rank tests. Analyses were performed using Prism (GraphPad Software). A similar experiment was performed to examine the *in vivo* efficacy of compound 80527891, except the infecting inoculum was 6.8×10^5^ cells of *C. albicans* strain SC5314.

We also performed a series of time-scheduled killings for determination of fungal organ burden. Briefly, one group of mice (*n*=16) was treated with compound 61894700 as above, whereas another group (untreated control; *n*=16) was injected with vehicle only. All animals were infected intravenously with 2.5×10^5^ *C. albicans* SC5314 cells and four animals from each group were then killed at 1, 2, 3 and 6 days post infection. For both treated and control (untreated) animals, fungal loads were determined by removing and homogenizing one kidney, the brain and the spleen from each mouse at time of being killed, and plating dilutions onto Sabouraud Dextrose agar (Becton Dickinson, Franklin Lakes, NJ, USA) plates containing ampicillin. After 24 h incubation colonies were counted to determine colony-forming units per gram of tissue. For analyses of fungal organ burden, colony-forming unit data were converted into logarithmic values and the nonparametric Mann–Whitney test was used to determine statistical significance between treated and untreated groups.

For histological analyses, kidneys were removed at the time of death or being killed, fixed in 10% buffered formalin, embedded in paraffin, sectioned and stained with Grocott–Gomori methenamine-silver before microscopic evaluation for determination of fungal morphology within infected tissues.

The *in vivo* efficacy of the 61894700 lead compound was also determined using the murine model of oropharyngeal candidiasis mostly as previously described^25,26^ with slight modifications. Groups of 6–8-week-old female BALB/c mice were immunosuppressed by subcutaneous injection with 225 mg/kg of cortisone acetate (Sigma-Aldrich) on day 1 pre-infection and days 1 and 3 post-infection. For infection, each mouse was anesthetized by an intramuscular injection with 50 μl of 2 mg/ml chlorpromazine chloride, and inoculated sublingually for 75 min with a swab saturated with 1×10^6^ *C. albicans* yeast cells (from cultures grown overnight in YPD broth at 25 °C) per ml of saline. For treatment, a 0.8-mg/ml solution of the test compound was prepared as above in saline for injection. One group of mice (*n*=5) received vehicle only and served as untreated control, and a second group of mice (*n*=5) received daily intraperitoneal injections of the compound in 0.5 ml of saline (for a dose equivalent to 20 mg/kg). In addition, the leading compound (prepared as above) was administered topically to a third group of mice (*n*=5) twice daily using a saturated cotton pad and daily swabbings of the whole oral cavity including the buccal mucosa, tongue, soft palate and other oral mucosal surfaces. Treatments started 2 days before infection. Macroscopic evaluation of the infection was used to generate a clinical score from 0 to 4 on the basis of the extent and severity of the tongue lesions,^26^ with a 0 denoting a healthy tongue surface and a 4 the most severe stage (white patches covering close to 100% of the surface). Scoring data were compared using the nonparametric Mann–Whitney test (one-tailed, based on the prediction that treatment will have beneficial effects, and assuming equal variances). After 3 days of infection, mice were killed, their tongues excised, embedded in paraffin, sectioned and stained with Grocott–Gomori methenamine-silver for visualization of fungal elements.

## Results and Discussion

### A large-scale chemical library screen identifies a series of small molecule inhibitors of *C. albicans* biofilm formation

**Figure 1:**
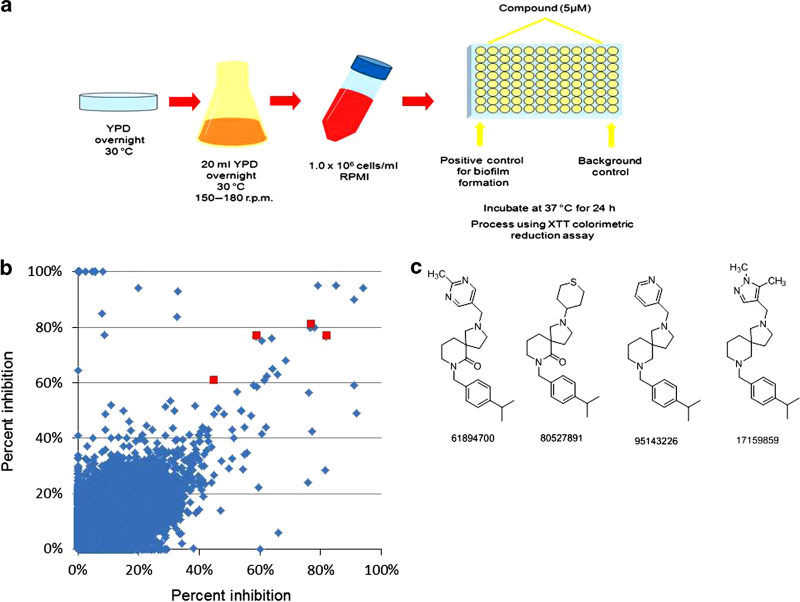
High-content screening identifies a novel structural series of small molecule inhibitors of *C. albicans* biofilm formation. (**a**) Diagram depicting the screening process, using the 96-well microtiter plate model of biofilm formation, that was employed to identify inhibitors of biofilm formation in *C. albicans* strain SC5314. Individual wells of the microtiter plates were seeded with fungal cells in the presence of a 5-μM concentration of each individual compound. The plates were incubated for 24 h at 37 °C to allow biofilm formation, and then assessed using the XTT-reduction metabolic assay, a semiquantitative colorimetric method to estimate the extent of biofilm formation. (**b**) Graphical representation of results from the primary screen for each of the 20,000 compounds from the NOVACore library. Results are expressed as percent inhibition compared with control biofilms formed in the absence of compound. Screening was performed in duplicate and the results of each screen are represented on the two axes. The red symbols indicate the four initial hits belonging to the same scaffold family of diazaspiro-decane compounds. (**c**) Chemical structures of the four bioactive compounds from this structural series identified in the primary screen.

There has been an increasing interest in developing novel approaches for the prevention and treatment of candidal biofilms,^13,15,27^ including the use of high-content/throughput screening techniques.^28–32^ In order to identify inhibitors of *C. albicans* biofilm formation we performed a primary screen using 20,000 small molecule compounds from the research-intensive, medicinally relevant NOVACore chemical library (Chembridge Corporation). As a screening assay, we employed the 96-well microtiter plate model of *C. albicans* biofilm formation originally developed by our group^20,21^ (Figure 1a). Our initial screen tested the effect of exposure to 5 μM of each of the 20,000 compounds from the library on *C. albicans* strain SC5314 biofilm formation, and was performed in duplicate (Figure 1b). This concentration was initially chosen on the basis of the fact that screening at medium to low concentrations normally identifies chemical matter that provides a more relevant biological (specific) starting point; this is in contrast to screening at higher concentrations that often leads to the identification of more numerous positive signals, many of which are typically of little value (that is, too much background noise/false positives).^33^ From these results we noticed that a particular scaffold family of diazaspiro-decane compounds was largely represented among the bioactive compounds. Several analogs from this series displayed biofilm-inhibitory activity, and some of the salient structural motifs within these hits were the para-isopropyl benzyl side chain off of the piperidine nitrogen, as well as an electron-deficient heteroaryl or heterocyclic ring off of the pyrrolidine nitrogen (Figure 1c). As shown in Table 1, this structural series is predicted to possess very favorable ‘drug-like’ physical chemical properties.

### Dose–response assays to establish potency and toxicity of the leading compounds

Confirmation of the biofilm-inhibitory activity of the hits within this series was performed via dose–response assays. Using the dose–response data, the IC_50_ for each compound, a measure of its potency, was determined (Table 1). In parallel to the determination of antibiofilm activity, we calculated the level of cellular cytotoxicity of the same compounds using an established assay in a human hepatocyte cell line. As shown in Table 1, the CC_50_ toxicity values are considerably higher than the effective concentrations necessary to inhibit biofilm formation, presumably indicating a good safety profile. On the basis of these characteristics, we selected compound 61894700 as the most promising lead candidate for further characterization.

### Secondary assays for subsequent characterization of the biofilm-inhibitory activity of the leading compound *in vitro*

**Figure 2:**
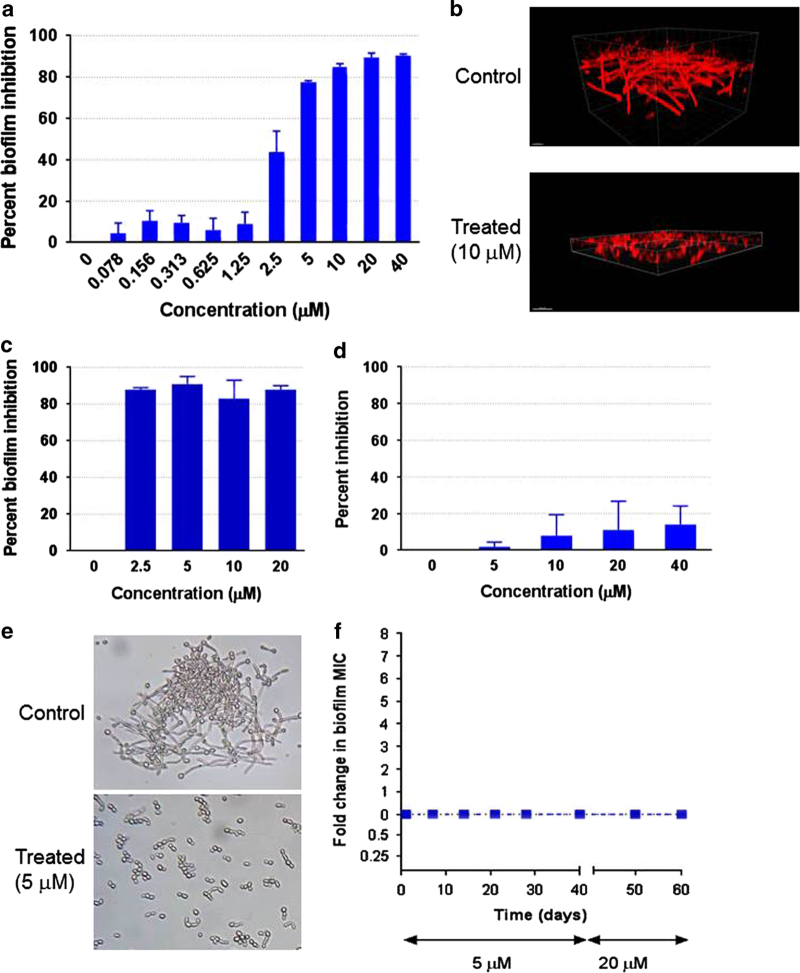
*In vitro* characterization of the 61894700 leading compound. (**a**) Dose-dependent inhibitory effects of compound 61894700 on *C. albicans* biofilm formation. Results are shown as the mean percent biofilm inhibition compared with control biofilms using the XTT assay. Experiments were conducted in duplicate, bars show s.d.’s. (**b**) Biofilms were formed in the presence or absence of this compound and stained with Concanavalin A-Alexa Fluor conjugate for CSLM visualization. Pictures show side views of the resulting biofilms. Bars=20 μm. (**c**) Biofilms were formed on silicone in the presence or absence of different concentrations of the compound. Percent inhibition was determined using the XTT assay. Experiments were conducted in duplicate, with results expressed as the means and s.d.’s. (**d**) Lack of activity against preformed *C. albicans* biofilms. Results are shown as the mean percent biofilm inhibition compared with control biofilms using the XTT assay. Experiments were conducted in duplicate, bars show s.d.’s. (**e**) Photomicrographs showing inhibition of *C. albicans* filamentation by compound 61894700 under strong filament-inducing conditions (YPD plus serum, 37 °C). Bars=20 μm. (**f**) Serial passage experiments demonstrate that growth in the presence of the lead compound does not induce resistance. Broth cultures of *C. albicans* SC5314 strain were established in YPD media containing 5 μM of 61894700—a concentration that inhibits filamentation and biofilm formation but does not have an effect on overall planktonic growth—and serial daily transfers performed for 40 days, after which time the concentration of compound was increased to 20 μM and experiments continued uninterrupted for an additional 20 days. The ability of the small molecule compound to still inhibit biofilm formation was determined using the same microtiter plate model methodology, with isolates recovered after the number of serial passages indicated.

We performed a number of secondary assays to further confirm and characterize the biofilm-inhibitory activity of the leading candidate compound. Figure 2a shows the detailed results for dose-dependent inhibitory effects on *C. albicans* biofilm formation for this leading compound. Confocal laser scanning microscopy indicated that incubation in the presence of this compound led to a decrease in biofilm thickness and cellular density as compared with biofilms formed in the absence of compound (Figure 2b). Moreover, as shown in Figure 2c, the lead compound displayed strong inhibitory activity against *C. albicans* biofilm development on silicone surfaces, a more clinically relevant model of biofilm formation on medical devices, particularly central venous catheters.^34^ In additional experiments we examined the *in vitro* activity of compound 61894700 against preformed *C. albicans* biofilms. As opposed to previous assays, for this set of experiments *C. albicans* SC5314 cells were allowed to form a fully mature biofilm on the wells of microtiter plates before treatment with the leading compound. Results indicated that preformed *C. albicans* biofilms were mostly recalcitrant to treatment with our leading compound (Figure 2d).

### The leading compound inhibits *C. albicans* filamentation but does not affect overall growth under planktonic conditions

Because the development of *C. albicans* biofilms is intimately linked with the fungus’ capacity to filament^35–37^ and filamentation represents a major virulence factor in its own right during candidiasis,^38–40^ we examined the ability of compound 61894700 to inhibit the yeast-to-hyphae transition. Under strong filament-inducing conditions resembling those encountered within the host (37 °C and serum),^41^ the leading compound inhibited hypha formation in a dose-dependent manner, as illustrated in Figure 2e and Supplementary Figure 1. This also confirmed that the compound retained its potency in the presence of serum, which is essential from a drug development point of view. Importantly, from this series of experiments it was also evident that the leading compound exhibited a marked effect on morphology but did not display an overall inhibitory activity on *C. albicans* growth. This was further corroborated by performing antifungal susceptibility testing under regular planktonic (free-floating) growth conditions, in which a minimum inhibitory concentration value for 50% inhibition could not be detected, even at much higher concentrations than those required to inhibit filamentation or biofilm formation (Supplementary Table 1). Thus, in contrast to conventional antifungals, our leading compound does not target cell viability; rather, it represents a true antivirulence compound, whose mechanism of action is inhibition of filamentation and biofilm formation, essentially disarming *C. albicans* of its two most important virulence factors during infection.^38,42,43^

### Repeated exposure to the lead compound does not lead to the development of resistance

Targeting virulence is particularly attractive in the case of antifungal drug development because it expands the extremely limited repertoire of potential targets in these eukaryotic organisms.^13,15^ Moreover, one additional advantage of antivirulence strategies is that they exert low levels of selective pressure and, thereby, should be less likely to foster resistance.^44^ Once we had established that our leading compound did not affect growth under planktonic conditions we performed a series of serial passage experiments to evaluate the potential to induce resistance upon repeated exposure to this small molecule, similar to previous experiments looking at development of azole resistance.^45^ In these experiments, experimental *C. albicans* populations were exposed to concentrations of this compound at which it inhibits filamentation and biofilm formation, but not overall planktonic growth: the initial concentration for the first 40 days was 5 μM, which was then increased to 20 μM for an additional 20 days. These experiments were performed using *C. albicans* strains SC5314 and 6482, a clinical strain with high propensity to develop resistance to multiple antifungals.^18,19^ We also performed these experiments under two different growing conditions, one that favors yeast growth (YPD medium in the absence of serum) and another that favors hyphal formation (RPMI 1640 medium). Cultures grown under filament-inducing conditions were monitored for hyphal formation before daily transfers, and there was no evidence that the compound had lost any of its filament-inhibiting activity throughout the length of experiments. In addition, when cells from populations evolved in the presence of drug and recovered at different time points during the experiments were used to develop biofilms, we did not observe any increase in the concentration at which compound 61894700 was able to exert its biofilm-inhibitory activity, as compared with the progenitors (Figure 2f and Supplementary Figure 2). Thus, we conclude that this antivirulence strategy is highly unlikely to foster the emergence of resistance.

### *In vivo* efficacy of the lead compound in clinically relevant murine models of invasive and oral candidiasis

**Figure 3:**
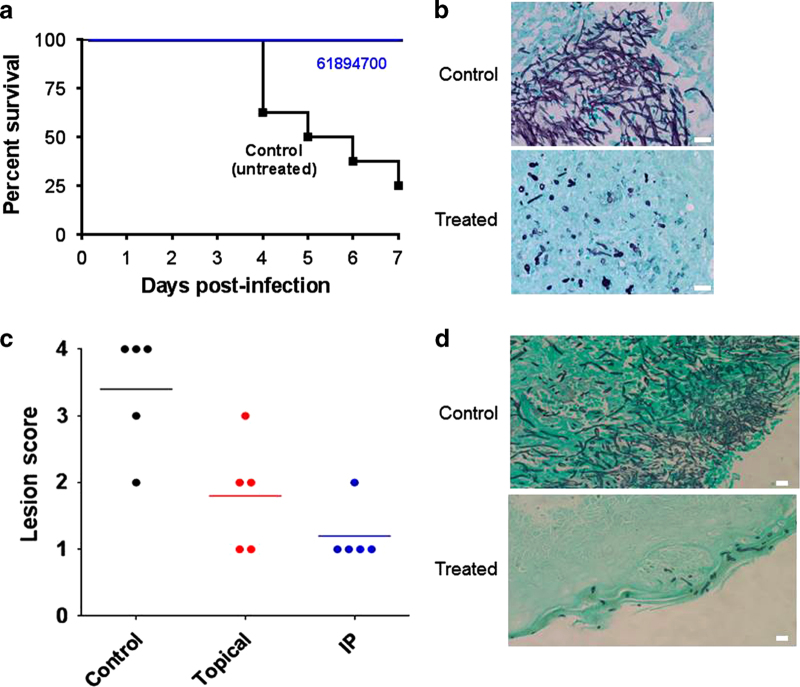
*In vivo* activity of the lead compound. (**a**) Protection in the murine model of hematogenously disseminated candidiasis. Compound 61894700 was administered to a group of mice (*n*=8) once daily by intraperitoneal injection at 20 mg/kg, starting 2 days before infection with *C. albicans* via the lateral tail vein. A control group of mice (*n*=8) received vehicle-only injections. Treatment continued for 7 days post infection. All treated mice survived the infection (*P*=0.0021 versus untreated), as determined by Kaplan–Meier log-rank tests. (**b**) Kidney sections from untreated mice showed characteristic kidney lesions of mostly filamentous nature, whereas isolated or small groups of mostly yeast cells were predominant in kidneys from treated mice. (**c**) Efficacy of compound 61894700 against oral candidiasis. Mice were inoculated orally with *C. albicans*. One group of mice (*n*=5) received vehicle only, whereas two additional groups (*n*=5 each) received treatment with compound 61894700 (20 mg/kg), starting 2 days before infection, either topically (twice daily) or systemically (once daily). The graph depicts the clinical score (from 0 to 4 on the basis of the extent and severity of the tongue lesions) for mice killed 3 days after infection. Statistically significant differences versus control were detected for animals treated topically (*P*=0.0406) or systemically (*P*=0.0108), using the one-tailed Mann–Whitney test. (**d**) Histology shows a filamentous biofilm covering the tongue surface and penetrating deep into the tissues of untreated animals, compared with fewer superficially located yeast cells in tongues from mice treated topically (similar observations were made in animals treated intraperitoneally). Bars=20 μm.

Given its promising *in vitro* characteristics, we next investigated the potential of the leading compound as a therapeutic, for which we used two clinically relevant models of candidiasis. The murine model of hematogenously disseminated candidiasis is standard in the field and routinely used for virulence studies and for testing the effects of established and new antifungal agents.^18,46–48^ Because of the mode of action (inhibition of filamentation and biofilm formation but lack of activity against preformed biofilms), we decided to use a pretreatment modality to mimic its use in a prophylactic antifungal regimen starting 2 days before infection, thus maximizing the chances to detect any protective effect. During the treatment period, all mice administered compound 61894700 survived the infection; this compared favorably with the mortality observed in mice in the untreated (vehicle-only) control group (Figure 3a). Consistent with its effect on fungal morphology, histological observations indicated that treatment with the compound inhibited filamentation *in vivo*, as kidneys (the main target organ)^49,50^ from treated animals contained mostly isolated yeast-like or partially elongated fungal cells as opposed to the typical filamentous lesions seen in tissue sections retrieved from untreated animals (Figure 3b). In addition, as expected because of its antivirulence mode of action with no direct effect on growth, no differences were observed in organ fungal burdens between untreated mice and mice treated with compound 61894700 when animals were killed at different times post-infection (Supplementary Figure 3), although a caveat here is that hyphal elements may have a lower plating efficiency than yeast cells, and therefore fungal burdens in organs from untreated mice may be underestimated. A second member of this structural series (compound 80527891) also demonstrated *in vivo* efficacy in this model of invasive candidiasis, further corroborating the drug-like characteristics associated with this scaffold family (Supplementary Figure 4). Finally, we examined the *in vivo* activity of the leading compound in the mouse model of oral candidiasis, which is associated with a biofilm etiology.^25,26^ The same prophylactic regimen as before was used to maximize chances of detecting protective effects. After inoculation, animals were closely monitored and assigned a clinical score based on signs of infection. Thick oral thrush, characterized by severe lesions in the tongue, was observed for untreated mice as infections progressed, whereas the severity of the lesions and associated clinical scores were significantly lower in animals treated either topically or systemically with compound 61894700 (Figure 3c). Histological examination of the tongues demonstrated a widespread biofilm and extensive lesions with numerous hyphae penetrating the epithelium of the tongues retrieved from untreated mice, whereas only scattered elongated yeast cells, mostly superficially located, were visible in tongues from mice treated with the compound (Figure 3d). Of note, this oral model uses immunosuppressed animals;^25,26^ thus, these results clearly indicate that an antivirulence approach may be successfully implemented for treating certain types of *Candida* infections, even in patients with compromised immune function.

In summary, our high-content screen identified a new structural series of antifungal compounds with a novel mode of action, namely inhibition of biofilm formation and filamentation. The top compound represents a promising candidate for the development of novel antivirulence approaches against *C. albicans* infections, which should minimize the development of resistance, and we are currently embarked on a medicinal chemistry campaign for lead optimization, as well as investigating its mechanism(-s) of action at the molecular level. As the need for new antifungals continues to grow because of dismal outcomes associated with fungal infections, a better understanding of fungal pathogenesis should yield novel targets for the development of novel antimycotic agents, which will both complement and add diversity to the currently exceedingly limited antifungal arsenal.

## Figures and Tables

**Table 1:** Identity, physicochemical properties and IC_50_ (potency) and CC_50_ (toxicity) values for the different small molecule compounds of the hit series with confirmed inhibitory activity against *C. albicans* biofilms

*NOVACore ID*	*IUPAC name*	*Formula*	*MW*	*Log P*	*p*K*a*	*tPSA*	*IC*_*50*_ *(μM)*	*CC*_*50*_ *(μM)*
61894700	2-[(2-methylpyrimidin-5-yl)methyl]-7-{[4-(propan-2-yl)phenyl]methyl}-2,7-diazaspiro[4.5]decan-6-one	C_24_H_32_N_4_O	392.537	3.45	8.66	49.33	2.70	104.10
80527891	7-{[4-(propan-2-yl)phenyl]methyl}-2-(thian-4-yl)-2,7-diazaspiro[4.5]decan-6-one	C_23_H_34_N_2_OS	386.594	3.96	10.6	23.55	3.36	62.70
95143226	7-{[4-(propan-2-yl)phenyl]methyl}-2-(pyridin-3-ylmethyl)-2,7-diazaspiro[4.5]decane	C_24_H_33_N_3_	363.539	4.21	9.64	19.37	4.59	81.42
17159859	2-[(1,5-dimethyl-1H-pyrazol-4-yl)methyl]-7-{[4-(propan-2-yl)phenyl]methyl}-2,7-diazaspiro[4.5]decane	C_24_H_36_N_4_	380.569	4.06	9.65	24.30	3.15	77.92

Abbreviation: PSA, topological polar surface area.
