# Supplementary material for: A novel small molecule inhibitor of Candida albicans biofilm formation, filamentation and virulence with low potential for the development of resistance
Source: NPJ Biofilms Microbiomes. 2015 Aug 12;1:15012–. doi: 10.1038/npjbiofilms.2015.12 (PMC4681527; doi:10.1038/npjbiofilms.2015.12)
Supplement: Supplementary Information [file npjbiofilms201512-s1.pdf]

Supplementary Figure 1. Dose-dependent inhibitory effects of compound 61894700 on *C. albicans* strain SC5314 filamentation assessed by microscopy. The compound was tested in serial 2-fold dilutions with concentrations ranging from to 0.039 to 40 µM under strong hyphal-inducing conditions (YPD plus 10% serum at 37°C).

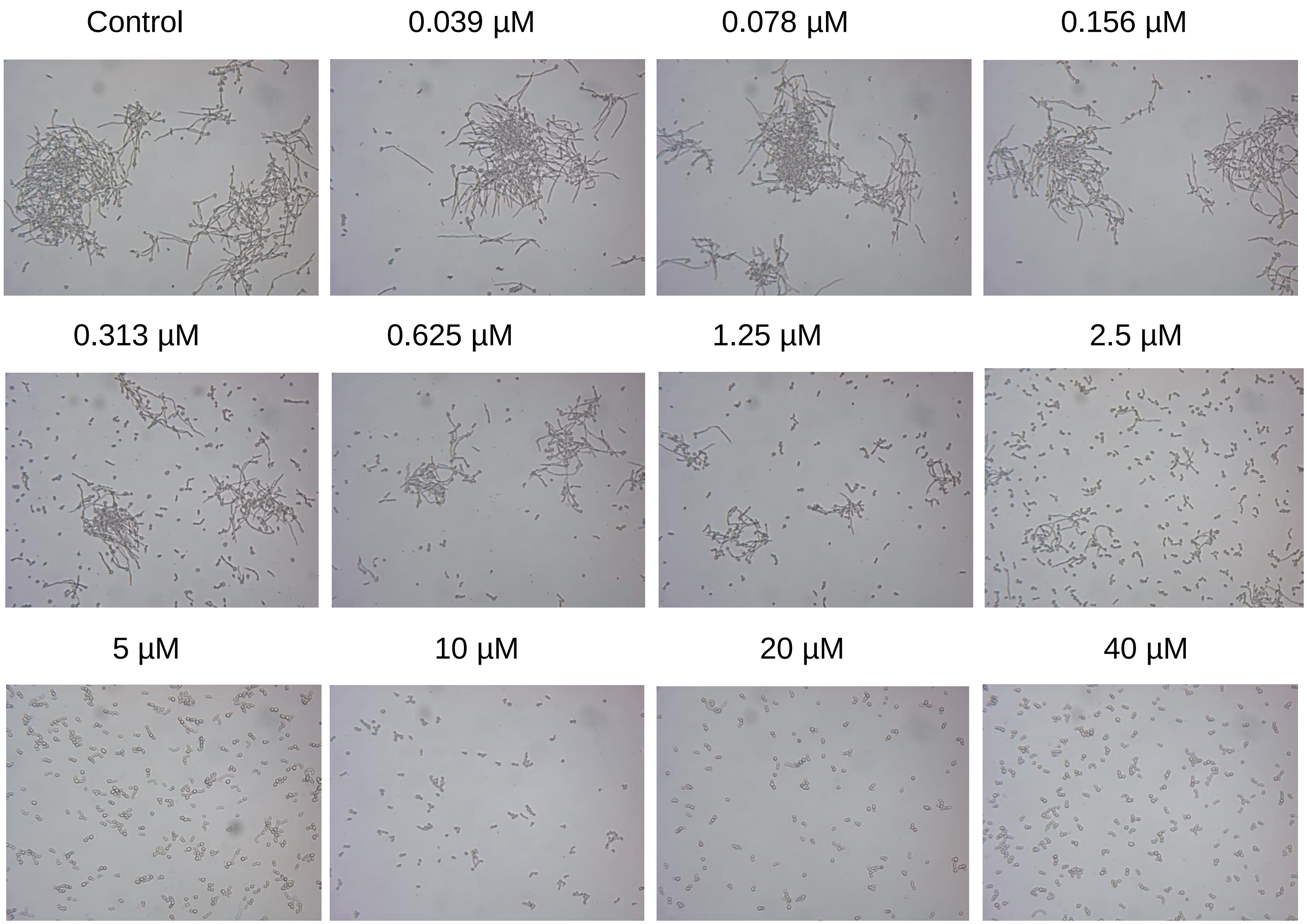

Supplementary Figure 2. Serial passage experiments demonstrate that growth in the presence of the leading compound for an extended period of time does not foster the development of resistance under different growth conditions and in two different strains of *C. albicans*. Broth cultures of *C. albicans* SC5314 and 6482 (a multi-drug resistant clinical isolate obtained from an HIV-infected patient with oropharyngeal candidiasis with high predisposition to develop resistance to multiple antifungal drugs) were established in media containing 5 μM of the 61894700 compound under both filament-inducing (RPMI) and non-inducing (YPD) conditions. Serial daily transfers were performed for 40 days; then the concentration of compound was increased to 20 μM for an additional 20 days. The ability of the small molecule compound to still inhibit biofilm formation was determined using the 96 well microtiter plate model methodology, with isolates recovered at different time points during the experiments. The graphs depict the fold change in sessile MIC values for each *C. albicans* strain and growth condition after the number of serial passages indicated.

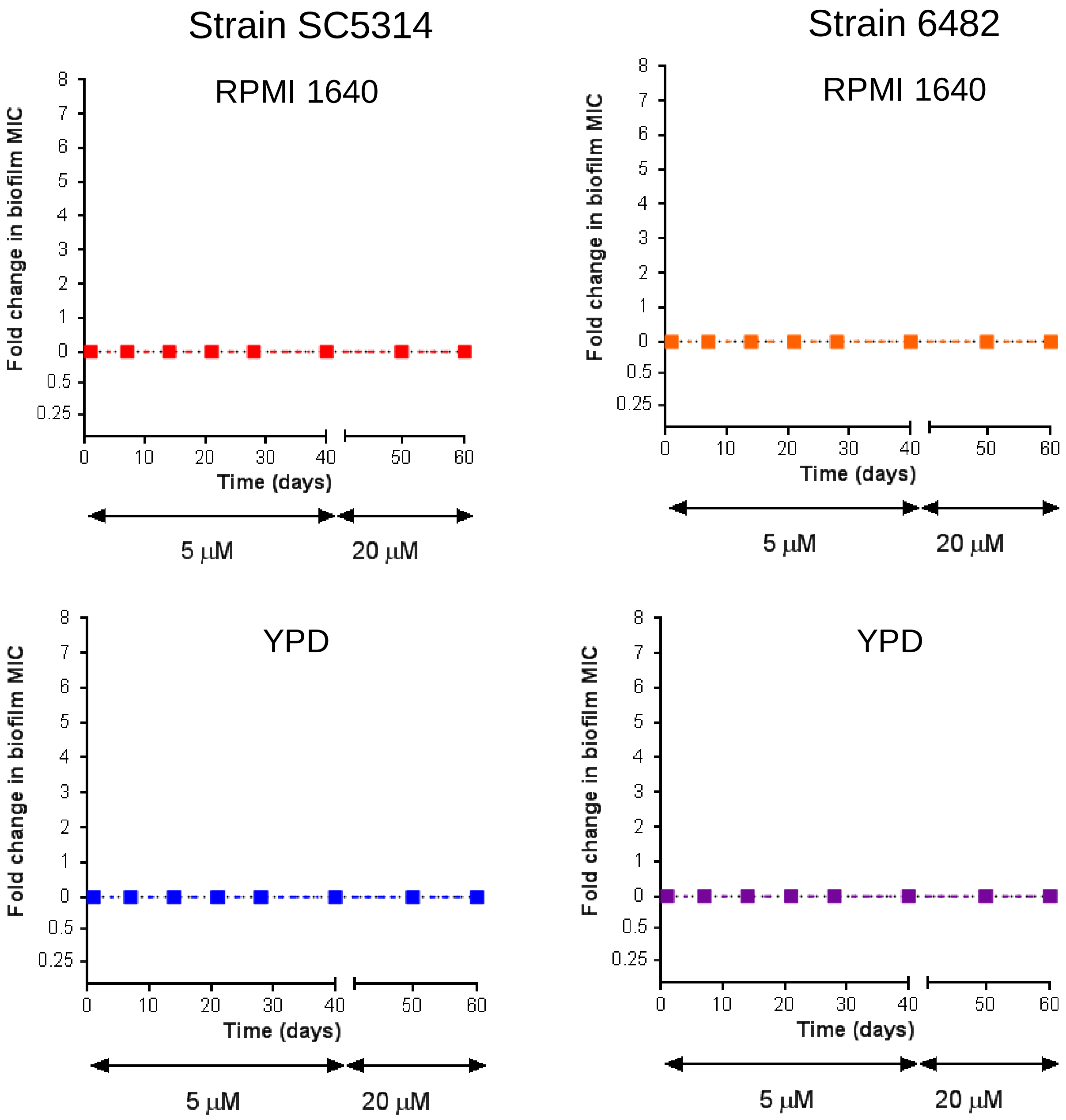

Supplementary Figure 3. Fungal organ burdens are similar in control (untreated) animals and in animals treated with the leading compound. One group of mice (n = 16) was treated with compound 61894700 (20 mg/kg, i.p., once daily starting two days prior to infection), whereas another group (untreated control) was injected with vehicle only. All animals were infected with 2.5 x 10^5 *C. albicans* SC5314 cells and four animals from each group were then sacrificed at 1, 2, 3, and 6 days post infection, at which time the fungal loads in brain, kidney and spleens were determined. Consistent with an antivirulence compound, there were no statistically significant differences in fungal organ loads in treated versus untreated animals as analyzed using the Mann-Whitney test.

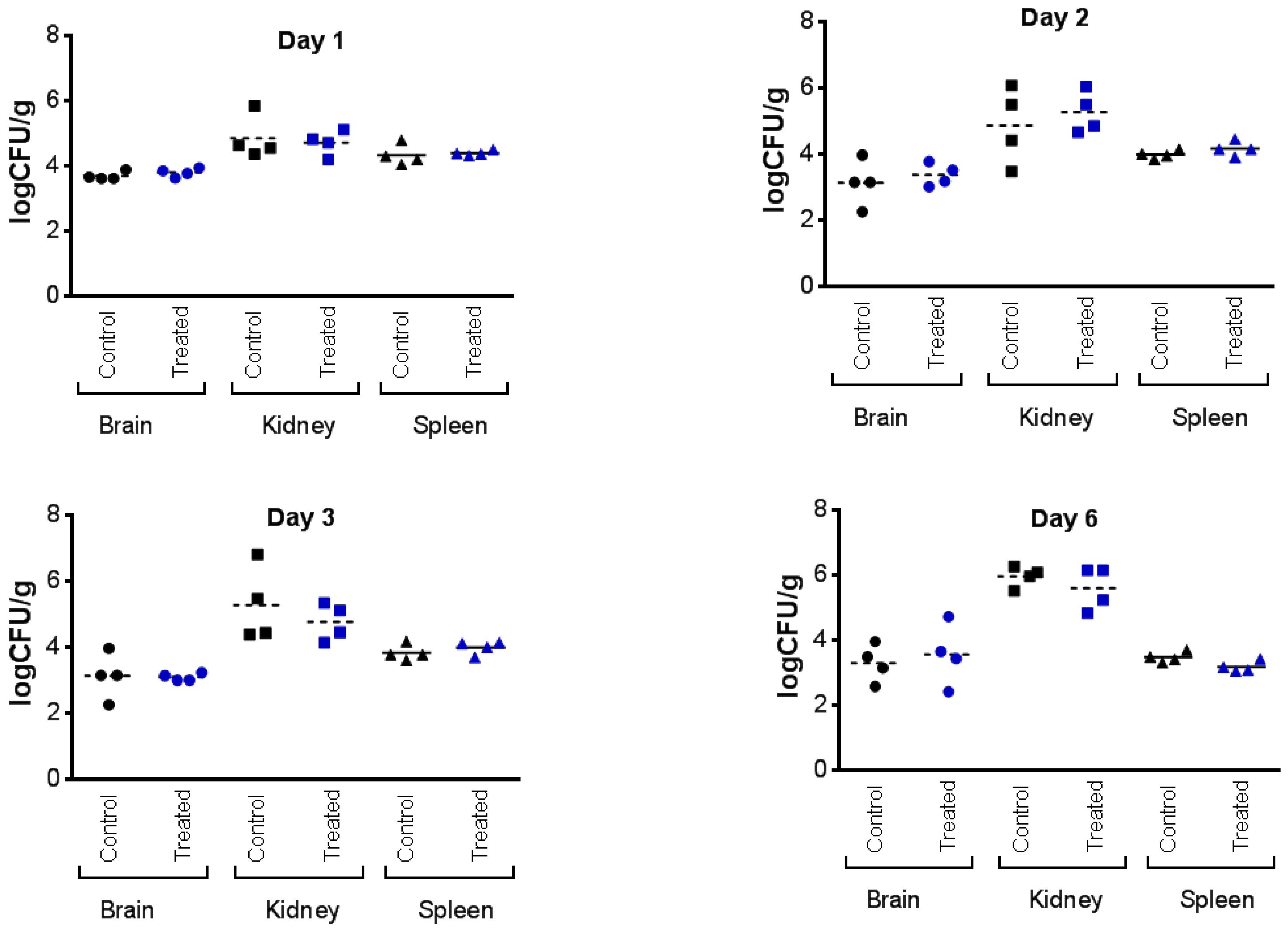

Supplementary Figure 4. *In vivo* activity of compound 80527891 in the murine model of hematogenously disseminated invasive candidiasis as assessed by survival proportions. Compound 80527891 was administered to a group of 6–8 week old female BALB/c mice (n = 8) once daily by intraperitoneal injection at 20 mg/kg, starting two days prior to infection with 6.8 x 10^5 cells of *C. albicans* strain SC5314 via the lateral tail vein. A control (untreated) group of mice (n = 8) received vehicle-only injections. Treatment continued for 7 days post infection. The resulting survival curves were analyzed using the Kaplan-Meier and logrank tests. (P = 0.0040 versus control/untreated).

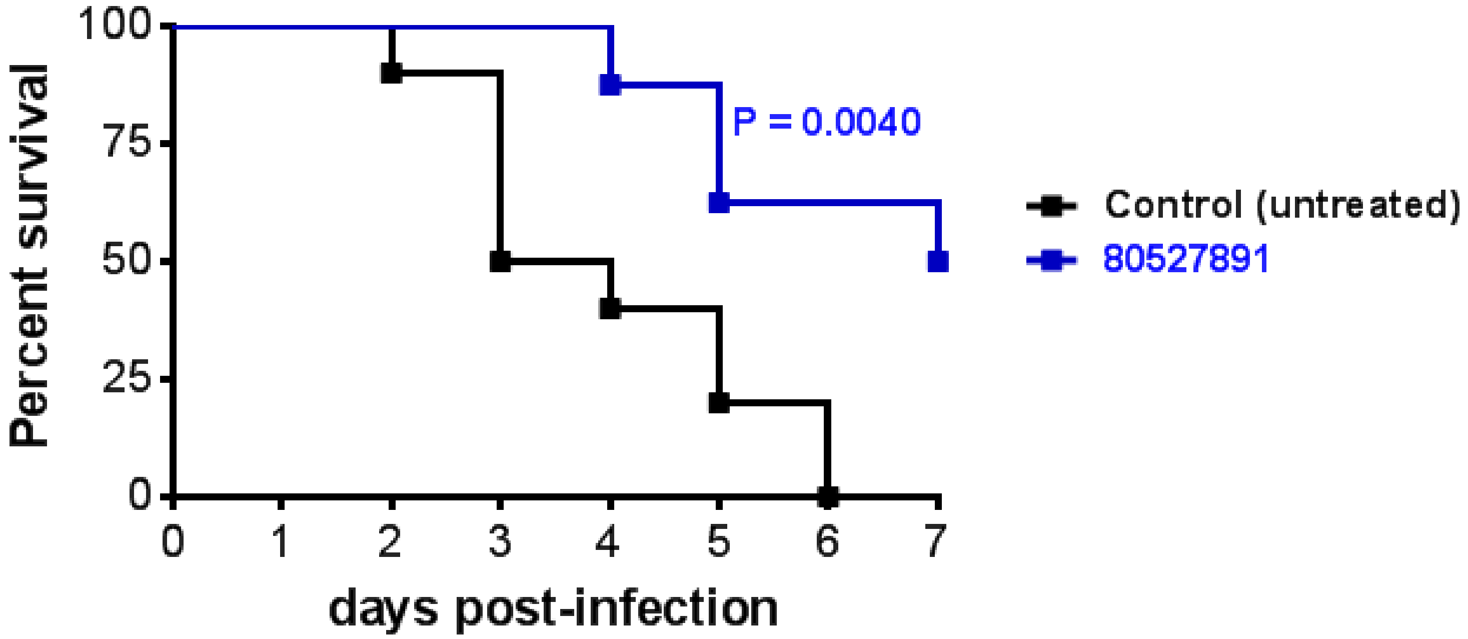

Supplementary Table 1. Results of antifungal susceptibility under planktonic conditions following CLSI broth microdilution techniques. Values are in μg/ml. AMB, amphotericin B; CSP, caspofungin, FLC, fluconazole.

Strain	AMB	CSP	FLC	61894700
C. albicans SC5314	1	1	≤0.125	>16
C. albicans 11-3478	0.5	0.5	0.25	>16
C. albicans 11-3479	0.5	1	1	>16
